# Nitric Oxide Regulates Plant Growth, Physiology, Antioxidant Defense, and Ion Homeostasis to Confer Salt Tolerance in the Mangrove Species, *Kandelia obovata*

**DOI:** 10.3390/antiox10040611

**Published:** 2021-04-16

**Authors:** Mirza Hasanuzzaman, Masashi Inafuku, Kamrun Nahar, Masayuki Fujita, Hirosuke Oku

**Affiliations:** 1 Molecular Biotechnology Group, Center of Molecular Biosciences (COMB), Tropical Biosphere Research Center, University of the Ryukyus, 1 Senbaru, Nishihara, Okinawa 903-0213, Japan; h098648@eve.u-ryukyu.ac.jp; 2 Department of Agronomy, Faculty of Agriculture, Sher-e-Bangla Agricultural University, Dhaka 1207, Bangladesh; 3 Department of Agricultural Botany, Faculty of Agriculture, Sher-e-Bangla Agricultural University, Dhaka 1207, Bangladesh; knahar84@yahoo.com; 4 Laboratory of Plant Stress Responses, Department of Applied Biological Science, Faculty of Agriculture, Kagawa University, 2393 Ikenobe, Miki-cho, Kita-gun, Kagawa 761-0795, Japan; fujita@ag.kagawa-u.ac.jp

**Keywords:** halophytes, antioxidants, reactive oxygen species, soil salinity, signaling molecules, abiotic stress

## Abstract

Facultative halophyte *Kandelia obovata* plants were exposed to mild (1.5% NaCl) and severe (3% NaCl) salt stress with or without sodium nitroprusside (SNP; 100 µM; a NO donor), hemoglobin (Hb, 100 µM; a NO scavenger), or Nω-nitro-L-arginine methyl ester (L-NAME, 100 µM; a NO synthase inhibitor). The plants were significantly affected by severe salt stress. They showed decreases in seedling growth, stomatal conductance, intercellular CO_2_ concentration, SPAD value, photosynthetic rate, transpiration rate, water use efficiency, and disrupted antioxidant defense systems, overproduction of reactive oxygen species, and visible oxidative damage. Salt stress also induced ion toxicity and disrupted nutrient homeostasis, as indicated by elevated leaf and root Na^+^ contents, decreased K^+^ contents, lower K^+^/Na^+^ ratios, and decreased Ca contents while increasing osmolyte (proline) levels. Treatment of salt-stressed plants with SNP increased endogenous NO levels, reduced ion toxicity, and improved nutrient homeostasis while further increasing Pro levels to maintain osmotic balance. SNP treatment also improved gas exchange parameters and enhanced antioxidant enzymes’ activities (catalase, ascorbate peroxidase, monodehydroascorbate reductase, and dehydroascorbate reductase). Treatment with Hb and l-NAME reversed these beneficial SNP effects and exacerbated salt damage, confirming that SNP promoted stress recovery and improved plant growth under salt stress.

## 1. Introduction

Among the plethora of abiotic stresses experienced by plants, salt stress has attracted the attention of plant scientists because of its complexity and its widespread effects over different regions of the globe. The global rise of temperature is gradually increasing the sea level and threatening coastal regions with salinity problems [1,2]. Salinity causing significant challenges in the way of crop cultivation and food production for a growing global population. Salt stress induces osmotic/dehydration stress and ion toxicity that disrupts the structure and function of biomolecules, including lipids, proteins, and DNA, thereby destroying the structure of biomembranes and causing damage to cell organelles [3]. These changes adversely affect vital phenological processes and disrupt critical physiological functions, including water uptake, water use efficiency, nutrient uptake and mobilization, transpiration, respiration, photosynthesis, and assimilation of photosynthate [4,5,6]. Current strategies to address salt stress have included incorporating modern cultivation practices and the introduction of plant species that can adapt to or tolerate salt stress.

*Kandelia obovata,* a mangrove species widely distributed in extremely saline environments in eastern Asia [7]. Learning from such halophytes and tailoring the traits associated with high salinity tolerance may build a foundation for salt tolerance in glycophytes.

Reducing photosynthesis is one of the major consequences of salt stress. Salt stress can damage chloroplast membranes and destroy the structure of these organelles. It also reduces stomatal conductance, thereby reducing carbon dioxide uptake, decreasing the carboxylation reaction of RuBisCO, and depressing photosystem II (PSII) activity, electron transport, and photophosphorylation activity [8,9,10]. This salt-induced stomatal closure and inhibition of photosynthesis exposes chloroplasts to excessive excitation energy that leads to the aggravated generation of reactive oxygen species (ROS), including superoxide (O_2_^•–^), hydrogen peroxide (H_2_O_2_), hydroxyl radical (OH^•^), and singlet oxygen (^1^O_2_) that cause serious damage to plant cells [11,12,13]. Plants have internal antioxidant defense systems that scavenge ROS and reduce this oxidative stress to some extent, and some plant species with highly active antioxidant systems are more tolerant of ROS and, therefore, of salt stress. Nonenzymatic antioxidants (ascorbate (AsA), glutathione (GSH), α-tocopherol, phenolic compounds, alkaloids, and nonprotein amino acids) and antioxidant enzymes [(superoxide dismutase (SOD), catalase (CAT), ascorbate peroxidase (APX), monodehydroascorbate reductase (MDHAR), dehydroascorbate reductase (DHAR), glutathione reductase (GR), glutathione peroxidase (GPX), glutathione *S*-transferase (GST), and peroxidases (POD)] function to scavenge ROS and decrease their levels under stress conditions [13,14,15].

Nitric oxide (NO) has recently been recognized as a potential signaling molecule and is often termed a plant hormone [16]. Enhancement of endogenous NO levels, as well as the exogenous application of NO, has been reported to improve salt tolerance via several different mechanisms. NO can stimulate the activity of proton pumps and the Na^+^/H^+^ antiport in the tonoplast, thereby improving the K^+^/Na^+^ ratio [17,18]. NO-induced improvement in the functioning of antioxidant systems also alleviates oxidative stress [19,20,21]. NO regulates the activity of phosphoenolpyruvate carboxylase kinase to mediate responses of plants to salinity [22]. NO also modulates stomatal conductance, promotes quenching of excess energy, and raises the quantum yield of PSII to improve photosynthesis [23,24,25,26].

Based on previous research, we hypothesized that NO could have a potential role in the tolerance of mangrove plants to high salinity. For this reason, we exposed mangrove plants to very high salt concentrations to understand the response and physiology behind the adaptative behavior of *K. obovata* growing in highly saline conditions. Our overall goal is to exploit this knowledge to instill salt tolerance in other cultivated plants that today face severe damage and crop losses due to salinity. Here, we examined mangrove plants’ response to high salt stress and determined the effects of exogenously applied NO on salt tolerance in this plant.

## 2. Materials and Methods

### 2.1. Growth Condition and Treatments

Healthy propagules were collected from the Iriomote mangrove forest, Okinawa, Japan. The propagules were washed and planted in Wagner pots containing salt-free sand. The propagules were allowed to grow in a glasshouse at the University of the Ryukyus, Okinawa, Japan, under controlled environmental conditions (relative humidity 60–70%, temperature 25 ± 2 °C, light 600 µmol m^−1^ s^−1^ and photoperiod from 12 to 14 h) in stagnant water. The experiment was conducted following a completely randomized design (CRD) with three replicates. After two months, the seedlings were exposed to freshwater or to water containing two salt levels (1.5 and 3% NaCl). Each set of seedlings was treated with 100 µM sodium nitroprusside (SNP, a NO donor), 100 µM hemoglobin (Hb, a NO scavenger), and 100 µm Nω-nitro-L-arginine methyl ester (l-NAME, a NO synthase inhibitor) and allowed to grow for two more months. The solutions were renewed every 15 days. At the end of the experiment, the plants were harvested and separated into shoots and roots. After washing well with distilled water, the plant parts were ground to a fine powder in liquid nitrogen and stored at −80 °C until further use. The experiment was replicated three times.

### 2.2. Quantification of Nitric Oxide

Nitric oxide was measured indirectly by quantifying nitrite. To determine the leaves’ NO contents, 0.5 g of freshly plucked leaves were ground in an ice-cold mortar with acetic acid buffer (50 mM, pH 3.6) and then centrifuged (10,000× *g*, 15 min) to remove the residue. The supernatants were decolorized with charcoal and mixed with Griess reagent. Griess reagent was prepared with N-1-naphthylethylenediamine dihydrochloride, sulfanilamide and phosphoric acid. The absorbance was taken at 540 nm, and the amount of NO was calculated using a standard curve [21].

### 2.3. Measurement of Gas Exchange and Photosynthetic Parameters

Data of stomatal conductance, intercellular CO_2_, photosynthetic rate, transpiration rate and water use efficiency were determined using a LiCOR 6400 open system portable infrared red gas analyzer (IRGA) (LI-COR Biosciences, Lincoln, NE, USA) from fully expanded leaves at 10 a.m. to 2.00 p.m. The conditions, which were used for the equipment/leaf chamber were as follows: ambient pressure 99.2 kPa, atmospheric CO_2_ concentration (Cref) 400 μmol mol^−1^, leaf surface area 6 cm^2^, PAR (Qleaf) was maximum up to 900–1000 μmol m^−2^ s^−1^ and the chamber water vapor pressure varied from 4.0 to 5.8 mbar [27]. SPAD values were recorded using a SPAD-502 m (Konica-Minolta, Tokyo, Japan).

### 2.4. Estimation of Proline Content

Proline (Pro) content was estimated by the method of Bates et al. [28]. Leaf was extracted by sulfosalicylic acid (3%) and glacial acetic acid, acid ninhydrin, and then the mixture was heated at the boiling water bath at 100 °C for 60 min. The reaction was terminated by placing the tube in an ice bath for 15 min. Toluene was then used as reaction reagents with the previous mixture. Upper toluene chromophore was used for spectrophotometric observation at 520 nm. The content of proline was calculated in the test sample using a standard curve.

### 2.5. Determination of Electrolyte Leakage

Electrolyte leakage (EL) was determined following the method of Dionisio-Sese and Tobita [29]. Electrical conductivity (EC) was measured by using a portable EC meter (Eutech Instruments, Singapore). Initial (EC_1_) and final (EC_2_) EC were recorded, and EL was determined by the following formula: EL(%) = (EC_1_/EC_2_) ×100.

### 2.6. Measurement of Lipid Peroxidation

The level of lipid peroxidation was measured by estimating malondialdehyde (MDA), a decomposition product of the per-oxidized polyunsaturated fatty acid composition of the membrane lipid, using 2-thiobarbituric acid (TBA) as the reactive material following the method of Heath and Packer [30]. The tissue was homogenized with ice-cold trichloroacetic acid (TCA) and then was centrifuged at 11,500× *g* at 4 °C. The supernatant was mixed with a reaction mixture of thiobarbituric acid (TBA) and TCA and heated in a water bath. Then the cooled supernatant mixture was read at 532 nm. The concentration of MDA was calculated by using the extinction coefficient of 155 mM^−1^ cm^−1^ and was expressed as nmol of MDA g^−1^ FW.

### 2.7. Measurement of Hydrogen Peroxide Generation

The amount of H_2_O_2_ generated was recorded using the method described by Yu et al. [31]. Fresh leaf samples of 0.5 g were extracted with 3 mL of potassium phosphate (K–P) buffer (50 mM, pH 6.5) and centrifuged at 11,500× *g* for 15 min to obtain a clear supernatant. A total of 2 mL of this supernatant was mixed with 666.4 μL of the reaction mixture (5.5 mM TiCl_4_ in 20% H_2_SO_4_), incubated for 10 min at room temperature and centrifuged again at 11,500× *g* for 12 min. To determine the H_2_O_2_ content, the absorption of the final supernatant was read at 410 nm with a spectrophotometer using 0.28 μM^−1^ cm^−1^ as the extinction coefficient, and the results are expressed as nmol g^–1^ FW.

### 2.8. Measurement of Antioxidant Enzyme Activities

Leaf samples were homogenized using potassium-phosphate (K-P) buffer (pH 7.0), AsA, β-mercaptoethanol, KCl, and glycerol as an extraction buffer. After centrifuging, leaf extracts were used to determine protein and enzyme activity. Soluble protein concentration was measured using Coomassie brilliant blue dye following the technique of Bradford [32]. The optical absorbance was then recorded at 595 nm, and a standard curve prepared by using bovine serum albumin was used for calculation.

Ascorbate peroxidase (EC: 1.11.1.11) activity was assayed following Nakano and Asada [33] by using K-P buffer (pH 7.0), AsA, ethylenediaminetetraacetic acid (EDTA), and H_2_O_2_ and absorbance were recorded at 290 nm. APX activity was calculated with an extinction co-efficient 2.8 mM^−1^ cm^−1^.

The activity of MDHAR (EC: 1.6.5.4) was measured by using a buffer solution containing ascorbate oxidase (AO), Tris-HCl buffer (pH 7.5), AsA, and nicotinamide adenine dinucleotide phosphate (NADPH) according to the method described by Parvin et al. [34]. Optical absorbance was observed at 340 nm. An extinction coefficient of 6.2 mM^–1^ cm^–1^ was used for calculating its activity.

Dehydroascorbate reductase (EC: 1.8.5.1) activity was assayed according to Nakano and Asada [33] by using dehydroascorbate (DHA), K-P buffer (pH 7.0), GSH, and EDTA and absorbance were recorded at 265 nm and extinction coefficient 14 mM^−1^ cm^−1^ was considered for calculating DHAR activity.

Catalase (EC: 1.11.1.6) activity was determined by using K-P buffer (pH 7.0) and H_2_O_2_ according to the method described by Hasanuzzaman et al. [35]. Optical absorbance was recorded at 240 nm and calculated using 39.4 M^–1^ cm^–1^ as the extinction coefficient.

Peroxidase (EC: 1.11.1.7) activity was estimated by using K-P buffer (pH 7), catechol, and H_2_O_2_ according to the method described by Gong et al. [36]. Absorbance was recorded at 470 nm and expressed as Umin^−1^ mg^−1^ protein.

Glutathione *S*-transferase (EC: 2.5.1.18) activity was assayed by using 1-chloro-2,4-dinitrobenzene (CDNB), Tris-HCl buffer (pH 6.5), and GSH according to the technique described by Rahman et al. [28] and absorbance was measured at 340 nm.

### 2.9. Measurement of Ion Contents

The contents of Na^+^, K^+^ and Ca^2+^ were measured from the dry shoot and root tissues by following Rahman et al. [37]. From ground homogenous dry plant samples, 100 mg was digested with the acid mixture (HNO_3_: HClO_4_; 5:1). Digestion of the sample was done at 70 °C for 48 h. The Na^+^, K^+^ and Ca^2+^ contents were determined from the digested solution by using the different lamps for the different minerals of atomic absorption spectrophotometer (AA-7000, Shimadzu, Japan), and the content was calculated using the standard curve of the respective mineral.

### 2.10. Statistical Analysis

Data accumulated from different parameters were subjected to analysis using CoStat v.6.400 [38] and one-way analysis of variance (ANOVA). For finding out mean differences among the replications, Tukey’s honest significant difference (HSD) test at the 5% level of significance was applied.

## 3. Results

### 3.1. Root and Shoot Length

**Figure 1:**
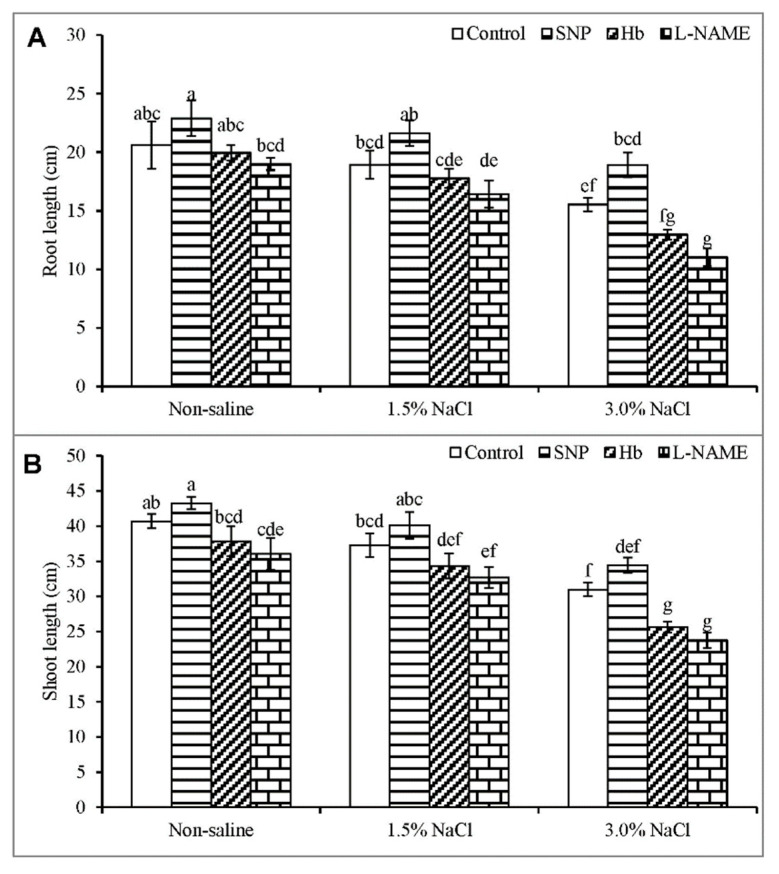
Root length (**A**) and shoot length (**B**) of *Kandelia obovata* treated with different salt concentrations and with 100 µM sodium nitroprusside (SNP, a NO donor), hemoglobin (Hb, a NO scavenger), or Nω-nitro- l-arginine methyl ester (l-NAME, a NO synthase inhibitor). Mean (± SD) was calculated from three replicates for each treatment. Bars with different letters are significantly different at *p* ≤ 0.05, applying Tukey’s HSD test.

Both root and shoot growth were significantly inhibited with the increase in NaCl concentration from 1.5% to 3%. Root length decreased by 8% of the control in response to 1.5% salt but was decreased by 25% by exposure to 3.0% NaCl (Figure 1A). Shoot length showed a similar pattern of reduction to increasing salt stress (Figure 1B). The inclusion of SNP in the salt medium somewhat alleviated the negative effect of salt stress, as indicated by an increase in root length of 14 and 22%, and an increase in shoot length of 8 and 11%, in plants growing in 1.5% and 3% NaCl, respectively (Figure 1A,B). The beneficial role of SNP was further established by treatment with Hb and l-NAME, as scavenging NO or inhibiting its production in plants under salt stress further exacerbated the decreases in root and shoot length, compared to the plants treated with salt alone (Figure 1A,B).

### 3.2. Endogenous Nitric Oxide Level

**Figure 2:**
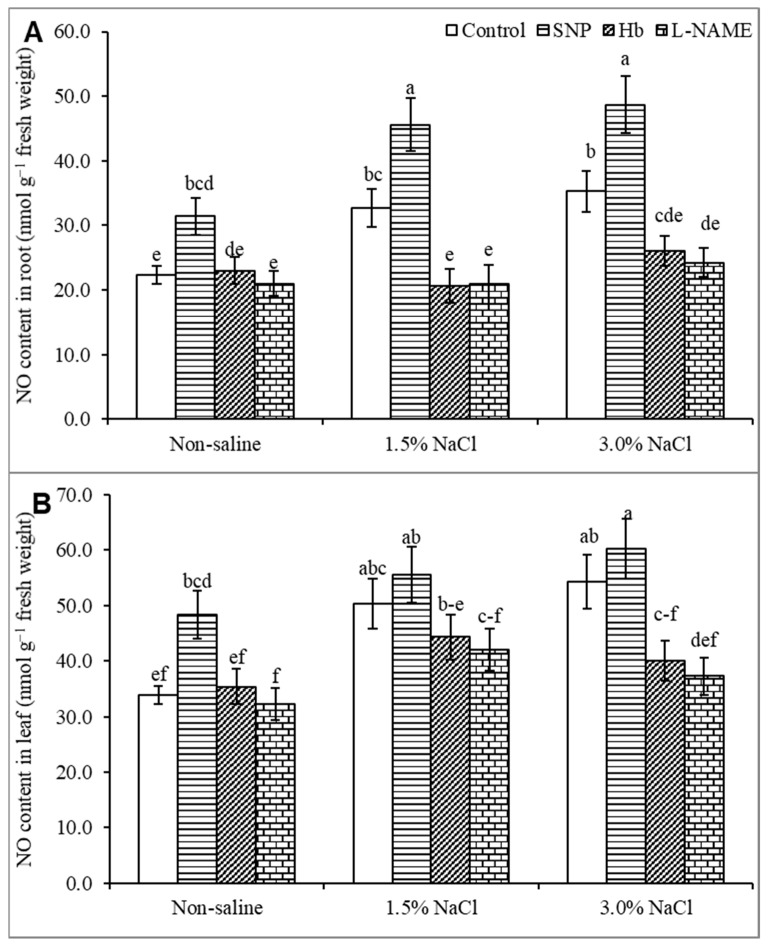
Endogenous NO content of *Kandelia obovata* root (**A**) and leaf (**B**) treated with different salt concentrations and with 100 µM sodium nitroprusside (SNP, a NO donor), hemoglobin (Hb, a NO scavenger), or Nω-nitro- l-arginine methyl ester (l-NAME, a NO synthase inhibitor). Mean (± SD) was calculated from three replicates for each treatment. Bars with different letters are significantly different at *p* ≤ 0.05, applying Tukey’s HSD test.

Treatment with SNP increased the NO content in nonstressed plants compared to the untreated control. Salt stress at 1.5% and 3% increased the NO content by 48 and 61%, respectively, in the roots (Figure 2A) and by 8 and 12%, respectively, in the leaf (Figure 2B), compared to the control treatment. Treatment of plants with SNP increased the NO content by 15% and 25% in the root (Figure 2A) and by 39% and 37% in the leaf (Figure 2B) in plants growing in 1.5 and 3% NaCl respectively, compared to salt stress alone. Treatment of salt-stressed plants with either Hb or l-NAME decreased the NO level drastically, both the root and the shoot, confirming the inhibitory effect of Hb or l-NAME on NO levels.

### 3.3. Gas Exchange and Photosynthetic Parameters

**Figure 3:**
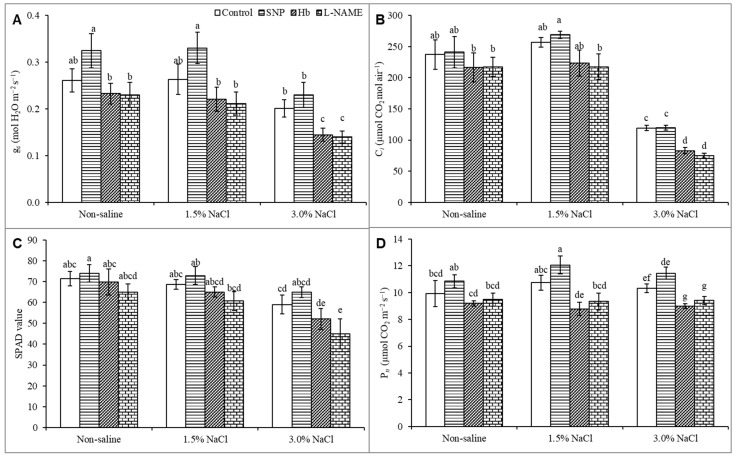
Stomatal conductance, g*_s_* (**A**); internal [CO_2_], C*_i_* (**B**); SPAD value (**C**) and net photosynthesis, P*_n_* (**D**) of *Kandelia obovata* treated with different salt concentrations and with 100 µM sodium nitroprusside (SNP, a NO donor), hemoglobin (Hb, a NO scavenger), or Nω-nitro- l-arginine methyl ester (l-NAME, a NO synthase inhibitor). Mean (± SD) was calculated from three replicates for each treatment. Bars with different letters are significantly different at *p* ≤ 0.05, applying Tukey’s HSD test.

Various photosynthetic parameters were affected by salt exposure. Stomatal conductance (g_s_) did not change under the mild 1.5% salt stress but was substantially reduced by 3% NaCl stress than the unstressed control (Figure 3A). Treatment of the salt-stressed plants with SNP increased g_s_, even in the plants treated with 1.5% NaCl (Figure 3A). Salt stress did not alter the *C_i_* value at 1.5% NaCl, but the *C_i_* value was decreased severely, by 50%, in the 3% salt treatment compared to the unstressed control. Treatment of the salt-stressed plants with SNP had no significant effect on the *C_i_* level than salt stress alone. However, treatment with Hb or l-NAME decreased the *C_i_* value drastically (Figure 3B). The SPAD value also decreased in response to 3% NaCl compared to the unstressed control. The SPAD value increased when salt-treated plants were treated with SNP, compared to plants exposed to salt stress alone. Treatment of salt-stressed plants with Hb or l-NAME substantially decreased the SPAD value (Figure 3C). As shown in Figure 3D, the P*_n_* was not affected significantly at 1.5% NaCl, but it was decreased by 24% under 3% NaCl stress compared to the unstressed control. Treatment of the salt-stressed plants with SNP somewhat alleviated the detrimental effects of salt stress, resulting in increased P*_n_* compared to the untreated salt-stressed plants. Treatment with Hb or l-NAME further decreased the P*_n_* value compared to salt stress alone, supporting a positive effect of NO on photosynthesis (Figure 3D).

### 3.4. Transpiration Rate and Water Use Efficiency

**Figure 4:**
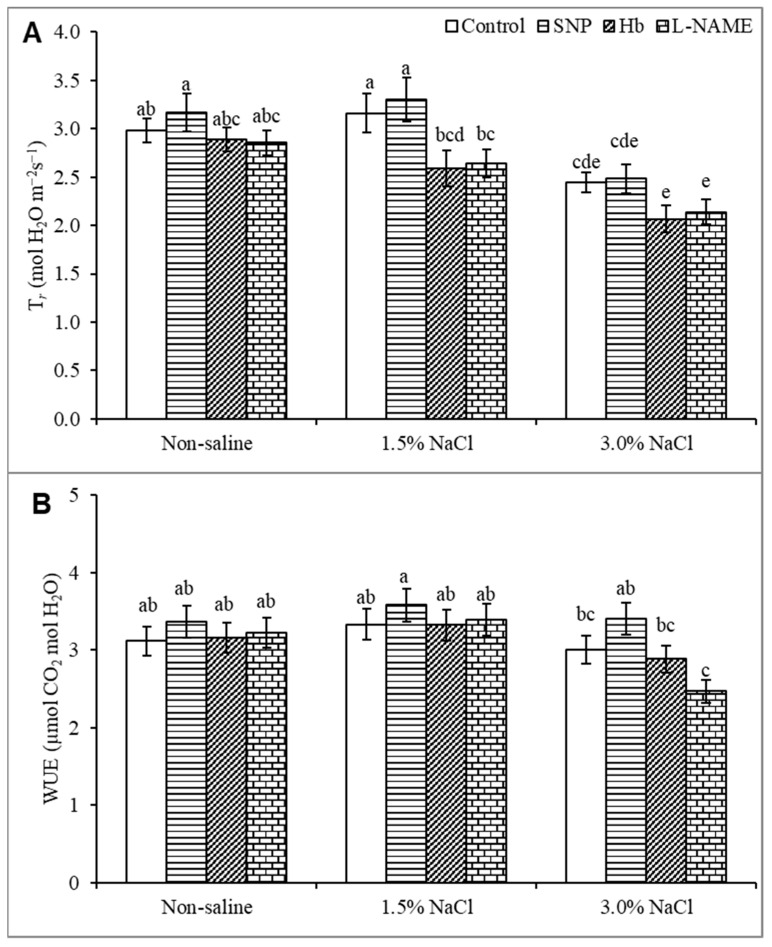
Transpiration rate (**A**) and water use efficiency, WUE (**B**) of *Kandelia obovata* treated with different salt concentrations and with 100 µM sodium nitroprusside (SNP, a NO donor), hemoglobin (Hb, a NO scavenger), or Nω-nitro- l-arginine methyl ester (l-NAME, a NO synthase inhibitor). Mean (± SD) was calculated from three replicates for each treatment. Bars with different letters are significantly different at *p* ≤ 0.05, applying Tukey’s HSD test.

The T*_r_* value was unaffected by treatment with 1.5% NaCl but was decreased by 20% in response to 3% NaCl stress compared to the unstressed control (Figure 4A). Treatment of salt-stressed plants with SNP did not significantly increase the T*_r_* value above that seen with salt stress alone. Treatment of salt-stressed plants with Hb or l-NAME further decreased T*_r_* (Figure 4A). The WUE value did not change under salt stress than the unstressed control, and treatment with SNP did not significantly increase WUE (Figure 4B). However, treatment with Hb or l-NAME substantially decreased WUE, indicating a role for NO (Figure 4B).

### 3.5. Proline Content and Electrolyte Leakage

**Figure 5:**
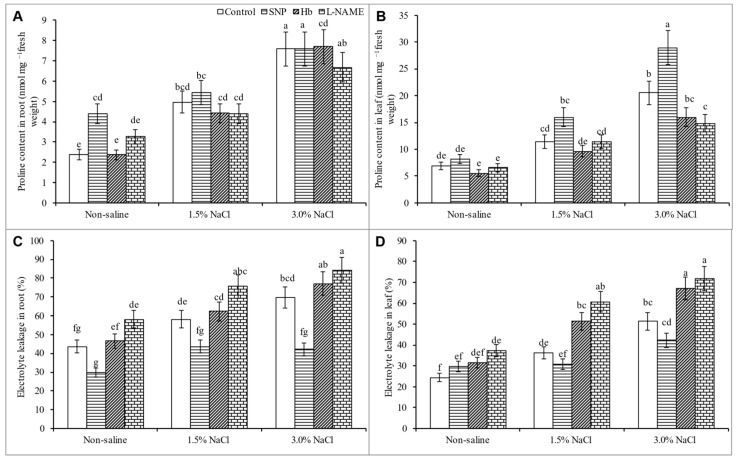
Proline content (**A**,**B**) and electrolyte leakage (**C**,**D**) in roots and leaves of *Kandelia obovata* treated with different salt concentrations and with 100 µM sodium nitroprusside (SNP, a NO donor), hemoglobin (Hb, a NO scavenger), or Nω-nitro- l-arginine methyl ester (l-NAME, a NO synthase inhibitor). Mean (± SD) was calculated from three replicates for each treatment. Bars with different letters are significantly different at *p* ≤ 0.05, applying Tukey’s HSD test.

The plant Pro content increased progressively with the increase in salt concentration. Compared to the control treatment, the Pro level increased by 66 and 197% in the leaf and by 104% and 217% in the root in response to 1.5 and 3% NaCl, respectively. Treatment of salt-stressed plants with SNP further increased the Pro content, whereas treatment with Hb or l-NAME decreased the Pro content in both the root and shoot (Figure 5A,B). Salt stress caused electrolyte leakage (EL) from the root and leaf, with EL levels increasing by 49% and 111% from the leaf and 33% and 60% from the root in response to 1.5% and 3% NaCl, respectively compared to the unstressed control. Treatment with SNP decreased EL from the salt-stressed plants., whereas treatment with Hb or l-NAME increased EL (Figure 5C,D).

### 3.6. Oxidative Stress Markers

**Figure 6:**
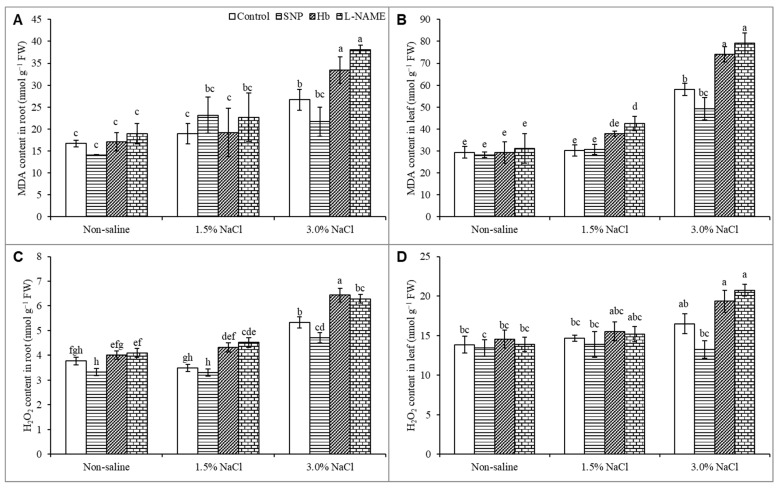
MDA content (**A**,**B**) and H_2_O_2_ content (**C**,**D**) in roots and leaves of *Kandelia obovata* treated with different salt concentrations and with 100 µM sodium nitroprusside (SNP, a NO donor), hemoglobin (Hb, a NO scavenger), or Nω-nitro- l-arginine methyl ester (l-NAME, a NO synthase inhibitor). Mean (± SD) was calculated from three replicates for each treatment. Bars with different letters are significantly different at *p* ≤ 0.05, applying Tukey’s HSD test.

The higher 3% NaCl level increased the MDA levels in both the leaf and root by 97% and 60%, respectively, compared to the unstressed control, whereas the lower 1.5% salt stress caused no significant change in MDA levels compared to the untreated control. Treatment with SNP decreased the MDA level in seedlings growing in 3% NaCl, whereas treatment with Hb or l-NAME caused increases in MDA levels in the salt-treated plants (Figure 6A,B). The hydrogen peroxide (H_2_O_2_) content was not significantly increased in the leaf or root in response to 1.5% NaCl stress and only showed a slight increase in response to 3% salt. Treatment of salt-stressed plants with SNP decreased the H_2_O_2_ levels compared to salt stress alone. Treatment of salt-stressed plants with Hb or L-NAME further increased the H_2_O_2_ levels above those observed in the SNP-treated salt-stressed plants (Figure 6C,D).

### 3.7. Activities of Antioxidant Enzymes

**Figure 7:**
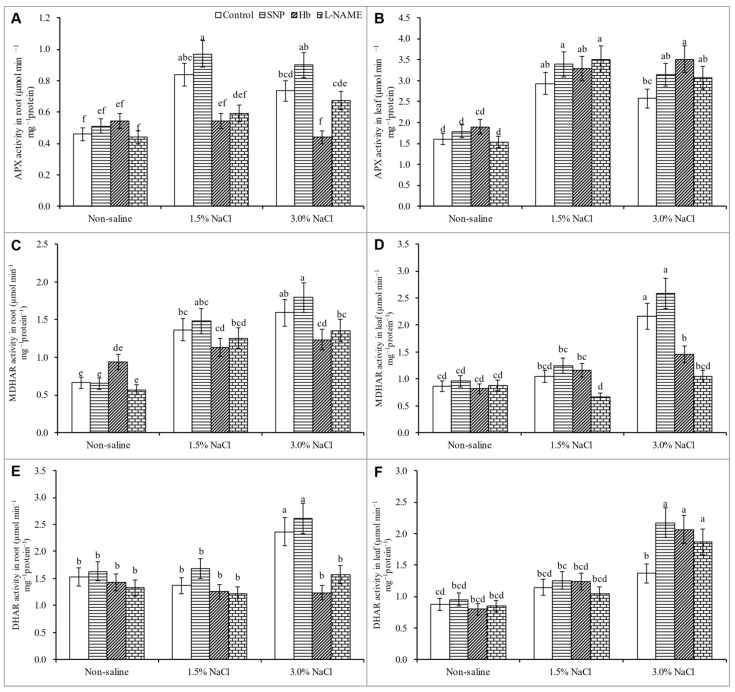
Activity of APX (**A**,**B**), MDHAR (**C**,**D**) and DHAR (**E**,**F**) in roots and leaves of *Kandelia obovata* treated with different salt concentrations and with 100 µM sodium nitroprusside (SNP, a NO donor), hemoglobin (Hb, a NO scavenger), or Nω-nitro- l-arginine methyl ester (l-NAME, a NO synthase inhibitor). Mean (± SD) was calculated from three replicates for each treatment. Bars with different letters are significantly different at *p* ≤ 0.05, applying Tukey’s HSD test.

In general, antioxidant enzyme activities (CAT, APX, MDHAR, and DHAR) increased in response to salt stress compared to the unstressed control. The exceptions were leaf and root DHAR activity in response to 1.5% NaCl stress and leaf MDHAR in response to both levels of salt stress. Treatment with SNP did not cause significant increases in enzyme activity in salt-treated plants, except for the leaf DHAR activity in the 3% NaCl treatment (Figure 7A–F).

**Figure 8:**
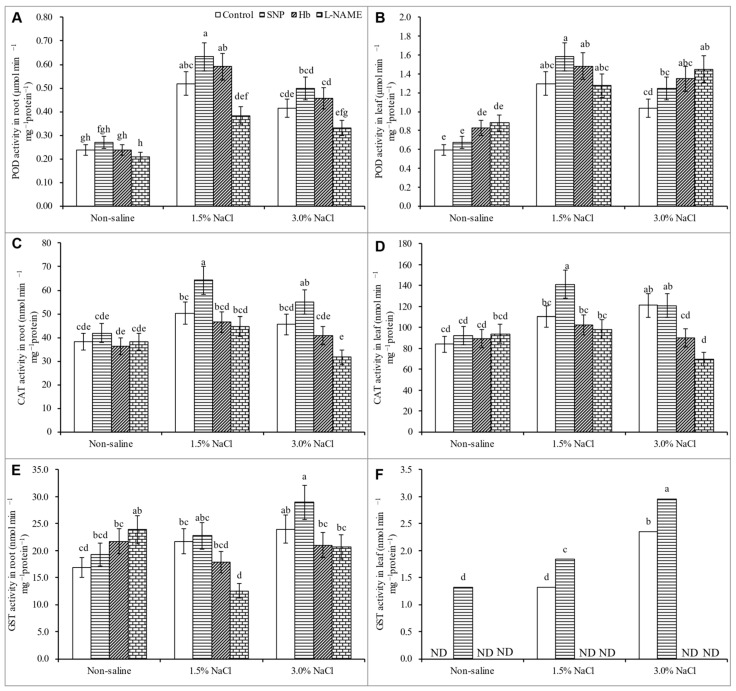
Activity of POD (**A**,**B**), CAT (**C**,**D**) and GST (**E**,**F**) in roots and leaves of *Kandelia obovata* treated with different salt concentrations and with 100 µM sodium nitroprusside (SNP, a NO donor), hemoglobin (Hb, a NO scavenger), or Nω-nitro- l-arginine methyl ester (l-NAME, a NO synthase inhibitor). Mean (± SD) was calculated from three replicates for each treatment. Bars with different letters are significantly different at *p* ≤ 0.05, applying Tukey’s HSD test.

The POD activity increased by 118 and 74% in the root and by 117% and 34% in the leaf in response to 1.5 and 3% NaCl treatment, respectively, compared to the unstressed control (Figure 8A,B). The activity of CAT increased only in response to 1.5% NaCl stress, compared to the unstressed control (Figure 8B,C). No changes were noted in root and leaf GST activity under salt stress compared to the unstressed control (Figure 8E,F). Treatment with SNP increased the CAT activity in the leaf and root above that observed in plants exposed to salt stress alone (Figure 8A–F).

### 3.8. Ion Contents

**Figure 9:**
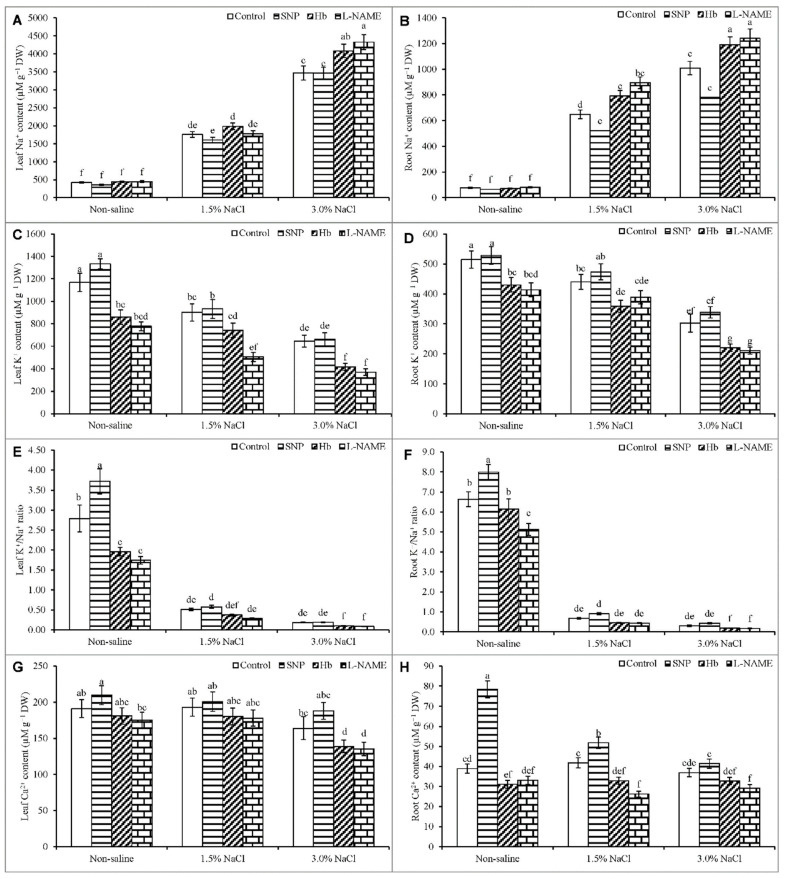
Na^+^ (**A**,**B**), K^+^ (**C**,**D**), K^+^/Na^+^ ratio (**E**,**F**) and Ca^2+^ content in roots (**G**) and leaves (**H**) of *Kandelia obovata* treated with different salt concentrations and with 100 µM sodium nitroprusside (SNP, a NO donor), hemoglobin (Hb, a NO scavenger), or Nω-nitro- l-arginine methyl ester (l-NAME, a NO synthase inhibitor). Mean (± SD) was calculated from three replicates for each treatment. Bars with different letters are significantly different at *p* ≤ 0.05, applying Tukey’s HSD test.

Salt stress created ion toxicity by increasing the plant Na^+^ content, decreasing the K^+^ content, and disrupting the Na^+^/K^+^ balance; however, it also disrupted nutrient homeostasis. Treatment of plants with 1.5% NaCl increased the Na^+^ levels in the leaf and root by 317% and 738%, respectively, while treatment with 3% NaCl increased these levels by 725% and 465%, respectively compared to the unstressed control (Figure 9A,B). By contrast, the K^+^ content in the leaf and root decreased by 23% and 14%, respectively, at 1.5% NaCl stress, and by 45% and 41%, respectively, at 3% NaCl compared to the unstressed control (Figure 9C,D). Consequently, the K^+^/Na^+^ ratio decreased considerably in both the leaf and the root (Figure 9E,F).

The content of Ca^2+^ decreased in response to 3% NaCl stress, whereas the root Ca^2+^ was not significantly affected compared to the unstressed control (Figure 9G,H). Treatment of salt-stressed plants with SNP decreased the Na^+^ content, increased the K^+^ content, and improved the K^+^/Na^+^ ratio in both the root and the leaf, compared to the plants treated with salt stress alone. The treatment with SNP also improved the Ca^2+^ level under salt stress, compared to salt treatment alone. Treatment with Hb or l-NAME caused further decreases in the Ca^2+^ level than the salt-stressed plants treated with SNP (Figure 9A–H).

## 4. Discussion

In this study, we investigated how the supplementation and inhibition of NO regulates the physiology of *K. obovate.* In salt-affected *K. obovata* plants, the endogenous NO level also increased, as reported in previous studies that have examined plants growing in stressful environments [39,40]. This rise in NO is suggested to play a signaling function in response to stress [16]. Treatment of salt-stressed *K. obovata* seedlings with SNP further increased the endogenous NO level, indicating an efficient uptake and accumulation of NO donated from SNP, in agreement with a similar role documented for SNP in previous studies [41,42]. By contrast, treatment with Hb and l-NAME decreased the NO levels both under normal and salt stress conditions, indicating the efficacy of these agents in scavenging NO or inhibiting its biosynthesis.

*Kandelia obovata* plants subjected to salt stress showed a significant decrease in shoot and root growth, which is due to the disruption of plant water relations that occurred from osmotic stress imposed by the salinity. Moreover, ion toxicity aggravates the stress response upon entry of the salt into the plant cells, which triggers secondary damage to the cell [43]. In the present study, NO supplementation via SNP treatment appeared to reinstate the plant growth suppressed by salt stress, while NO removal by scavenging with Hb or suppression of NO production with l-NAME prevented this restoration and worsened the effects of salt stress in *K. obovata* plants. A similar effect of NO on the restoration of plant growth under salt stress also has been demonstrated by other researchers [25,40,44,45,46,47].

In this study, *K. obovata* showed substantial changes in several gas exchange and photosynthetic parameters, namely *P_n_, g_s_, T_r_, C_i_,* and WUE, especially when exposed to 3% NaCl. Salt stress creates a physiological drought through the generation of osmotic stress. This physiological drought can cause stomatal closure, thereby reducing photosynthetic CO_2_ assimilation. In the halophyte *K. obovata*, a high salt stress level caused a decrease in *g_s_* and a subsequent marked decrease in *C_i_*. As facultative halophyte, *K. obovata* plant was able to tolerate 1.5% salt and did not show any changes in P*_n_* under that level of stress; however, its P*_n_* value decreased in response to growth in 3% salt compared to the unstressed control. Several reasons may explain this response. In the present study, *g_s_, T_r_, C_i_*, and WUE decreased at the higher level of salt stress. Reductions in *g_s_* and *C_i_* under high salt stress are the direct reasons for the observed decrease in *P_n_*. The salinity-induced decline in *P_n_* was positively related to the reduction in *g_s_* and *C_i_*, as reported previously in soybean plants [48].

Treatment with Hb and L-NAME reversed the positive effects of SNP in the absence of salt stress. In *K. obovata* plants, the negative effects on the SPAD value, *P_n_, g_s_, T_r_, C_i_*, and WUE were more prominent following treatment with the NO inhibitors. Treatment of the salt-treated plants with Hb and l-NAME caused a further decrease in SPAD value, *P_n_, g_s_, T_r_, C_i_*, and WUE than was observed with salt stress alone. These inhibitors hindered NO accumulation in two ways, by scavenging and by suppressing the NO biosynthesis that would be produced by plants under salt stress. Inhibition of uptake and accumulation of NO from exogenous sources like SNP would also be suppressed.

Plants facing osmotic stress accumulate osmolytes inside the cells to correct their water balance. The large increase in Pro, which increased gradually with the rise in salt level, indicates that *K. obovata* can cope with salt-induced osmotic stress. This capacity was further increased by SNP treatment, as indicated by the further increase in Pro content. Proline is widely known for its osmoprotective effects, as well as its proficiency at scavenging hydroxyl radicals and stabilizing the structure and function of macromolecules, including DNA, protein, and membranes [48,49,50]. Proline protects the photosynthetic machinery and thus enhances photosynthesis. It acts as an energy storage material during salt stress, thereby improving survival and adaptation [45,51]. Treatment of salt-stressed *K. obovata* plants with SNP increased the Pro level, thereby improving osmoregulation, ROS scavenging, and the stabilization of biomembranes and biomolecules. In the present study, exogenous SNP promoted an increase in the Pro level in salt-affected plants, as previously reported for Pro levels in cadmium-stressed mung bean [52]. The decrease in Pro following Hb and l-NAME treatments that decreased NO availability also abruptly decreased the Pro level, indicating a potential role for SNP in regulating Pro levels.

Salinity is similar to all other abiotic stresses in that it causes oxidative stress, and salt-induced oxidative stress has been reported in different plant species [4,53]. The resulting oxidative damage is a consequence of altered photosystem activity and stomatal movement, ion toxicity, disrupted nutrient homeostasis and disturbed antioxidant defense mechanisms in the salt-affected plants [44,54,55]. In our study, salt-induced oxidative damage was observed in *K. obovata* plants, with the more severe damage noted at the higher salt level. The high levels of H_2_O_2_ measured in this plant confirmed a salt-induced ROS overproduction. The high MDA levels are the result of membrane lipid peroxidation due to salt-induced oxidative damage. Some reports support similar findings in other plants under salt stress [44,54].

In the present study, SNP decreased the levels of H_2_O_2_ and MDA in the salt-affected plants. Previous studies on mung bean have shown that SNP treatment can induce inhibition of lipoxygenase (LOX) activity and reduce oxidative stress [52]. Nitric oxide can react with O_2_^•−^ to form peroxynitrite (ONOO^−^), which can then be detoxified by peroxiredoxins [56]. Treatment with SNP reduced the production of H_2_O_2_ and O_2_^•−^ and led to a reduction in MDA accumulation in ryegrass under copper toxicity stress [19]. Nitric oxide lessens oxidative damage in several ways, through reduction of O_2_^•−^ and subsequent oxidative stress, through inhibition of LOX activity, and through NO-induced regulation of molecules like Pro, which also scavenge ROS and stabilize biomembranes and biomolecules.

The results from Hb and l-NAME treatments in the present study confirm that oxidative damage was exacerbated to an even greater extent by these NO modifiers to even higher levels than were observed by salt stress alone. Treatment of salt-stressed plants with Hb and l-NAME promoted the production of H_2_O_2_, raised MDA levels, and increased electrolyte leakage above the levels seen in the salt-stressed, SNP-treated plants. This result confirmed that Hb and l-NAME reduced NO levels, thereby promoting greater oxidative damage.

In the present study, both leaf Na^+^ and root Na^+^ contents were strongly increased to levels expected to create ion toxicity [18,57]. The increased Na^+^ levels decreased K^+^ levels, and decreased K^+^/Na^+^ ratios in both the leaf and root indicate a condition of disrupted and imbalanced ion homeostasis. These cations directly compete with each other to enter the plants under salt stress and represents a common sign of salt-induced ion toxicity, as demonstrated in several previous studies [44,46,58]. The leaf and root Ca^2+^ contents also decreased similarly to the K^+^ contents, in agreement with the findings of other studies [44,46]. Treatment of salt-stressed plants with NO in the form of SNP resulted in a decreased root and leaf Na^+^ content, an increased K^+^ content, an increased K^+^/Na^+^ ratio, and an increased Ca^2+^ content, compared to salt stress alone. The NO provided by SNP promoted cell membrane reconstruction and restored the ability to exclude toxic ions and to take up nutrient elements necessary for plant development [44]. Treatment of cotton plants with SNP upregulated the expression of vital salt-tolerant genes, including the plasma membrane Na^+^/H^+^ antiporter (*SOS1*) and the vacuolar Na^+^/H^+^ antiporter (*NHX1*), allowing salt-stressed plants to decrease Na^+^ uptake from the salt solution, while simultaneously promoting an increased K^+^ uptake [46]. Several other studies have also shown an SNP-induced reduction of Na^+^ uptake and augmentation of K^+^ uptake, with an eventual increase in the K^+^/Na^+^ ratio [44,58].

Some evidence also supports our findings regarding SNP-induced improvement in nutrient uptake. For example, the concentrations of minerals in the leaves and roots of salt-affected wheat plants were enhanced by treatment with SNP [18]. Improved K^+^ levels in salt-affected cotton were also demonstrated in response to SNP treatment [44,58,59,60]. In the present study, *K. obovata* plants treated with Hb and l-NAME showed reduced endogenous NO levels under salt stress but higher levels of Na^+^, lower levels of K^+^ and Ca^2+^ and a reduced K^+^/Na^+^ ratio in both roots and leaves. These results indicate that Hb and l-NAME removed NO and inhibited its accumulation, thereby promoting the development of ion toxicity and disruption of mineral homeostasis that was even more severe than were observed under the salt-stress condition. Hb and l-NAME inhibited the endogenous production of NO in salt-affected plants and also eliminated the beneficial effect of exogenous SNP in salt-stressed *K. obovata* plants.

Nitric oxide enhances the transformation of O_2_^•−^ to H_2_O_2_ and O_2_ through enhanced activities of SOD; the H_2_O_2_ is then detoxified by the H_2_O_2_ scavenging enzymes APX, GPX, and GST [59]. The activities of the enzymes MDHAR and DHAR, which are AsA recycling enzymes, were increased by treatment with SNP. Therefore, SNP treatment most likely increased the levels of AsA, allowing greater scavenging of ROS. Treatment with SNP was reported to increase GR’s activity, which increased the GSH content by enhancing its recycling process, and this increased GSH also participated in ROS detoxification processes [21,59]. Treatment with SNP improved the activities of SOD, POD, and CAT in wheat plants subjected to salt stress [44], in agreement with the present findings regarding antioxidant defense systems, ROS detoxification, and oxidative stress alleviation by SNP. Treatment of salt-stressed plants with Hb or l-NAME reversed these responses, leading to a reduction in antioxidant enzyme activities and an enhancement of oxidative stress. These findings further confirm a role for NO in enhancing the antioxidant defense system and oxidative stress tolerance.

## 5. Conclusions

The facultative mangrove plant, *K. obovata,* showed the least damage under low-level salt stress (1.5%). This low stress did not change the studied physiological attributes significantly, thereby confirming the salt tolerance of this plant species. At the higher level of salt stress (3%), the plants showed oxidative damage. When the salt-affected plants were treated with SNP, the endogenous NO level increased, and the plants showed significant improvements in most of the studied physiological attributes compared to salt-treated control plants. The effect of NO was further confirmed by reducing the NO levels in salt-stressed plants with the NO scavenger Hb and the NO biosynthesis inhibitor l-NAME. The use of these NO modulators exacerbated the adverse effects of salt stress, providing strong evidence for a role for NO in improving salt adaptation and tolerance traits in the *K. obovata* plant.

## Data Availability

Not applicable.
